# The sliding cupping therapy as an alternative strategy for treating plaque psoriasis: A randomized controlled trial

**DOI:** 10.1097/MD.0000000000048111

**Published:** 2026-03-20

**Authors:** Jia Liu, Shun Guo, Jing Tan, Miao Miao, Yue Shi, Cheng Tan, Yuegang Wei

**Affiliations:** a Department of Dermatology, Affiliated Hospital of Nanjing University of Chinese Medicine, Jiangsu Province Hospital of Chinese Medicine, Jiangsu, China.

**Keywords:** narrowband ultraviolet B, plaque psoriasis, psoriasis area and severity index, sliding cupping intervention

## Abstract

**Background:**

The sliding cupping therapy represents a traditional Chinese medicine therapy and receives much appreciation for treating plaque psoriasis. This study was designed to test the hypothesis that sliding cupping therapy is non-inferior to narrowband ultraviolet B (NBUVB) therapy in improving disease severity in patients with plaque psoriasis.

**Methods:**

This prospective study recruited 60 patients with plaque psoriasis who were randomized to receive either sliding cupping intervention or NBUVB treatment. The cup was moved 30 times for each skin lesion until the target skin area turned purple. The initial dose (mJ/cm^2^) of ultraviolet radiation b (UVB) was determined based on sun-reactive skin types I through VI, which ranged from 300 mJ/cm^2^ to 800 mJ/cm^2^. Both treatments were performed 3 times per week for 8 weeks. The primary endpoint was the percentage reduction in Psoriasis Area and Severity Index (PASI) score at week 8, with secondary endpoints, including Physician’s Global Assessment (PGA), body surface area, visual analogue scale scores, and quality of life measures.

**Results:**

The total response rates were 69.23% (18/26) and 79.17% (19/24) for patients receiving sliding cupping intervention and those receiving NBUVB treatment, respectively, which showed no significant difference (*P* = .526). The PASI scores, body surface area, and PGA were reduced in patients with plaque psoriasis at W0, W4 and W8 after either sliding cupping intervention or NBUVB treatment (*P* <.001), and these reductions were not significantly different between the patients receiving sliding cupping intervention and those receiving NBUVB treatment at W0, W4, W8, and W12. At W8, the mean percentage reduction in PASI was 62.4% (95% CI, 54.9–69.8) in the sliding cupping group and 66.9% (95% CI, 59.6–74.2) in the NBUVB group, with no significant difference between groups. The total response rates were 69.23% (18/26) and 79.17% (19/24), respectively (*P* = .526). Patients receiving sliding cupping intervention and those receiving NBUVB treatment did not show statistically significant differences in these outcomes at W0, W4, W8, and W12 (*P* >.05).

**Conclusion:**

The overall results suggest that sliding cupping therapy exhibits statistically similar efficacy and safety profiles as NBUVB treatment, especially at 8 weeks after treatment.

## 1. Introduction

Psoriasis is a lifelong papulosquamous skin disease that is attributed to epidermal hyperproliferation, abnormal differentiation of keratinocytes, as well as intensive infiltration of immune cells.^[1]^ The global incidence of psoriasis increased by 26.53% from 1990 to 2019 and significantly affects nearly 2% to 3% of people worldwide.^[2,3]^ Psoriasis consists of disproportional 4 clinical types, including plaque (88.1%), guttate (6%), erythrodermic (4.6%) and sebopsoriasis (0.9%).^[4]^ Chronic plaque psoriasis represents the most common subtype of psoriasis and is characterized by erythematous plaques covered with silvery-white scales, significantly leading to poor life quality for the affected individuals.^[5]^ Plaque psoriasis is a complex interplay between genetic predisposition, such as the presence of a well-recognized risk allele of HLA-C*06:02, and environmental triggers such as obesity, smoking, and alcohol consumption.^[6,7]^ The current treatment methods available for psoriasis predominantly cover topical agents (topical corticosteroids, vitamin D analogue, or calcineurin inhibitor), phototherapy [narrowband ultraviolet B (NBUVB)], traditional systemic drugs (etretin and immunosuppressants), and biologic disease modifying antirheumatic drugs (DMARDs).^[8]^ A large proportion of patients with psoriasis require long-term therapy, and thus the desired treatment goals are not only short-term disappearance of clear psoriasis plaques but also lasting remissions over time.^[9]^ Long-term use of high-potency topical agents in normal-appearing skin or intertriginous areas may lead to skin atrophy, telangiectasia, striae, skin irritation, burning, and pruritus. The adverse effects of phototherapy involve erythema, pruritus, blistering, photoaging, and photocarcinogenesis.^[10]^ Emerging biological agents, such as tumor necrosis factor-alpha (TNF-α) inhibitors, IL-17 inhibitors, and IL-12/23 blockade, have developed with a long-term maintained control of psoriasis compared to conventional treatments, while various side effects, and disease relapse after drug withdrawal are challenging for the clinical implementation of these treatments.^[11,12]^ Recently, complementary and alternative medicine therapies have been developed for dermatology, among which cupping receives much attention as a complementary and alternative medicine therapy for treating psoriasis.

Cupping has been employed for at least 2000 years among Asian and Middle Eastern countries to deal with several medical disorders including blood disorders, inflammatory conditions, pain relief, physical relaxation, and mental well-being.^[13]^ This technique is performed by placing one of several kinds of dome-shaped cups (primarily made of bamboo, glass, or earth) over a painful area or acupuncture point on the skin to create a suction (negative pressure) inside the cup by means of either an air pump device or flaming heating power.^[14]^ The negative pressure formed within the cup can lift the tissue to make the local place hyperemia or hemostasis, creating a massage-like effect and thus achieving desired treatment goals in human diseases including stroke and hypertension.^[15–17]^ In addition, the sliding cupping therapy is widely applied in the treatment of various skin disorders in China. Acne belongs to the disorders of appendages which affects the face, neck, shoulders, chest, and back.^[14]^ It has been reported that wet cupping can relieve the skin breakouts of acne.^[18]^ Moreover, the safety and effectiveness of cupping therapy have also been confirmed in the treatment of subcutaneous disorders like neurodermatitis and chronic urticaria.^[19,20]^ More importantly, cupping therapy has been found to relieve several adverse effects related to chemotherapy and offer a better quality of life in colorectal cancer patients.^[21]^ There are mainly 4 types of cupping methods, including retained cupping, flash cupping, sliding cupping, as well as needling cupping.^[22]^ The sliding cupping used to treat plaque psoriasis received much appreciation in China due to its safety and cost-effectiveness.^[23]^ A previous study compared the efficacy of the sliding cupping in treating plaque psoriasis but setting the sliding cupping placebo as control instead of current treatment methods available for psoriasis.^[24]^ Therefore, there is still a lack of high-quality medical evidence demonstrating the efficacy of the sliding cupping. The purpose of the study is to analyze efficacy and safety of sliding cupping therapy for the treatment of plaque psoriasis compared to NBUVB treatment, so as to facilitate decision making regarding plaque psoriasis treatment.

## 2. Methods

### 2.1. Participant selection

Participants were consecutively recruited among patients with plaque psoriasis who visited dermatology outpatient clinics of the Jiangsu Provincial Hospital of Traditional Chinese Medicine between September 2020 and July 2022. Inclusion criteria: an investigator-confirmed diagnosis of plaque psoriasis according to the 2018 China College of Dermatology diagnostic criteria for plaque psoriasis, specifically plaque-type psoriasis involving ≤ 15% of total body surface area (BSA), psoriasis area and severity index (PASI) ≥12, and static physician’s global assessment (sPGA) ≥3 at their first screening visits^[25]^; disease duration >6 months prior to screening; and aged 18 to 65 years at the time of screening visit. Exclusion criteria: erythrodermic psoriasis, pustular psoriasis, guttate psoriasis, drug-induced psoriasis; other active skin lesions (such as eczema) which potentially interfere with the assessment of treatment outcomes; the use of biological agents such as TNF inhibitors in the previous 8 weeks, IL-17A antagonists within the previous 12 weeks, or IL-12/IL-23 antagonists in the previous 12 weeks, or the in-take of trial-related drugs in the previous 3 months before screening; any ongoing evidence of local or systemic acute and chronic infection at screening; uncontrolled or unstable health conditions at screening, including hepatic, renal, respiratory, neoplastic, or endocrine diseases; abnormal laboratory values at screening, including, but not limited to, a serum AST or ALT concentration >1.5-fold of the average, red blood cell count, hemoglobin level, platelet count, and white blood cell count lower than the normal limit; women being pregnant, breastfeeding, or planning pregnancy during the study period; a history of excessive drinking or drug abuse; and a history of psychiatric abnormalities. Participants were randomly assigned in a 1:1 ratio to the sliding cupping or NBUVB group using a computer-generated random number sequence with a fixed block size of 4. The randomization sequence was generated by an independent statistician who was not involved in participant recruitment, treatment administration, or outcome assessment. Allocation concealment was ensured using sequentially numbered, sealed, opaque envelopes prepared in advance according to the randomization sequence. After confirmation of eligibility and completion of baseline assessments, the treating physician opened the next envelope in sequence to determine group assignment. Outcome assessors and data analysts were not involved in the randomization process. All patients signed written informed consent before their participation. The study protocols fulfilled the Declaration of Helsinki and received the approval from the Ethics Committee of the Jiangsu Provincial Hospital of Traditional Chinese Medicine.

### 2.2. Treatment protocols

Participants eligible for the study were randomized to receive either sliding cupping intervention or NBUVB treatment, and a computer-generated random sequence was conducted by independent statisticians to determine assignment to treatment arms. Sliding cupping intervention: after shielding the eyes with a black opaque mask, participants were requested to expose their skin lesions in the lying position. Once disinfected, the skin lesion areas were applied by using liquid paraffin. The cotton ball was soaked with 95% ethanol, ignited, and then put inside the upside-down cup using tweezers. The cup with negative pressure was immediately placed on the skin lesion area, with the skin pumped 3 to 4 cm up. The operator held the skin surrounding the cup in 1 hand (left), and moved the cup repeatedly along the skin lesion area using another hand (right). The cup was moved 30 times for each skin lesion until the target skin area turned purple. NBUVB treatment: the initial dose (mJ/cm^2^) of UVB was determined based on sun-reactive skin types I through VI^[26]^ or a recommended dosing schedule according to the manufacturer’s instructions: the participants with skin type I and II received 300 mJ/cm^2^, those with skin type III and IV received 500 mJ/cm^2^, and those with skin type V and VI received 800 mJ/cm^2^. The duration (second) of UVB was determined by the dose/intensity. Before starting NBUVB treatment, the participants should wear UV goggles and shorts. When NBUVB exposure was determined and the electric fan was on, the participants were instructed to stand in the center of the phototherapy cabin, with both upper limbs holding. During the NBUVB exposure, the participants were allowed to stop the treatment in the case of burning and tingling sensations. At subsequent exposures, the dose was increased by 20% per session in the case of no evidence of an erythematous response and by 10% per session if an erythematous response was barely perceptible following 48 hours. No dose increase was delivered in the case of persistent asymptomatic erythema. The NBUVB exposure was interrupted until recovery in the case of presence of a well-defined painful erythematous response. The maximum exposure dose was set as 2000 mJ/cm^2^ for skin type I and II, 3000 mJ/cm^2^ for skin type III and IV, and 5000 mJ/cm^2^ for skin type V and VI. The dose was unchanged if there was 4 to 7 days of delay from the last exposure, the dose was resumed with 75% of the last dose if there was 1 to 2 weeks of delay from the last exposure, and the dose was resumed with 50% of the last dose if there was 2 to 3 weeks of delay from the last exposure. If there was 3 to 4 weeks of delay from the last exposure, the dosing schedule started from the beginning. Two treatment arms were performed 3 times per week for 8 weeks and the patients were followed up at an interval of 12 weeks.

### 2.3. Outcome measurements

The primary outcome was evaluated by a reduction (%) of PASI scores after 8 weeks of treatment. The PASI scores combines the severity of 3 clinical signs of psoriasis lesions, erythema, infiltration, and desquamation, at 4 anatomical sites.^[27]^ The maximum PASI score is 72, and higher scores reflect greater psoriasis severity. Clinical recovery: the skin lesions almost disappeared or only a few slight skin lesions remained, with more than 95% reduction of PASI score (PASI95) and the pruritus disappeared. Excellent response: the pruritus was significantly reduced, with 70% to 94% reduction of PASI score. Good response: the pruritus was reduced, with 30% to 69% reduction of PASI score. No response: the pruritus failed to be reduced, with <30% reduction of PASI score.

The secondary outcomes were PASI, PGA, BSA, Visual Analogue Scale (VAS) scores for lesion pruritus, dermatology life quality index (DLQI) score, and patient-reported quality of life (PRQoL). The PGA scale is a 5-point tool representing the overall degree of clinical signs of psoriasis lesions.^[28]^ The BSA covered with psoriasis is estimated by the number of fingerprints on the skin lesions on a body part (%). The DLQI is a questionnaire containing 10 questions with total scores ranging from 0 to 30, which was employed to assess the health-related quality of life with regard to skin diseases. A higher score is indicative of more influence on the quality of life.^[29]^ The questions 1 and 2 focus on “Symptoms and feelings,” the questions 3 and 4 focus on “Daily activities,” the questions 5 and 6 focus on “Leisure,” the questions 7.1 and 7.2 focus on “Work or school,” the questions 8 and 9 focus on “Personal relationships,” and the question 10 focuses on “Treatment.” The VAS is an assessment tool that is employed to examine lesion pruritus ranging from 0 mm (no pruritus) to 100 mm (maximum pruritus) at each visit.^[30]^ All PASI and PGA assessments were performed by trained dermatologists who were blinded to treatment group allocation and were not involved in treatment administration or randomization. For categorical efficacy analysis, “total response” was predefined as the proportion of patients achieving at least a good response, including clinical recovery, excellent response, and good response (i.e., ≥30% reduction in PASI score). The total response rate was analyzed as a secondary outcome and was not used as the primary efficacy endpoint. For consistency with psoriasis clinical trial reporting standards, categorical PASI response rates were additionally evaluated. PASI75, PASI90, and PASI100 were defined as ≥75%, ≥90%, and 100% reduction in PASI score from baseline, respectively. The proportions of patients achieving PASI75, PASI90, and PASI100 at week 8 and week 12 were analyzed as secondary outcomes. Adverse events (AEs) were assessed at each treatment visit and follow-up visit through active inquiry by investigators using a standardized safety checklist, supplemented by spontaneous patient reporting. All AEs were recorded regardless of suspected causality and were evaluated for severity and relationship to the study intervention. Serious AEs were predefined as events resulting in death, life-threatening conditions, hospitalization, or significant disability.

### 2.4. Statistical analysis

The Shapiro–Wilk test was utilized to determine whether continuous data fit the normal distribution. In the case when the normal distribution was satisfied, continuous data were obtained using mean ± standard deviation (s.d.). For continuous data failing to satisfy normality in the independent *t*-test, median (InterQuartile Range, 25%–75%) was obtained. Difference between the sliding cupping and NBUVB groups was assessed by using the unpaired *t*-test or the Mann–Whitney U test. Difference for data at baseline (W0) to 4 weeks (W4), 8 weeks (W8), and 12 weeks (W12) was determined by using the one-way analysis of variance (ANOVA) or Kruskal-Wallis test, and these differences were compared between the sliding cupping and NBUVB groups by using the 2-way ANOVA. Categorical data were obtained using frequency (percentage) and compared by using the Fisher exact test. The sample size was determined based on a non-inferiority design with the primary endpoint defined as the percentage reduction in PASI score at week 8. Based on previous studies of NBUVB therapy for plaque psoriasis, a mean PASI reduction of approximately 65% with a standard deviation of 18% was assumed. A non-inferioritymargin of 15 percentage points was predefined as the largest clinically acceptable difference. With a 2-sided significance level (α) of 0.05 and a statistical power of 80%, a minimum of 22 patients per group was required. Assuming an anticipated dropout rate of approximately 10% to 15%, the target enrollment was set at 25 to 26 participants per group. All efficacy analyses were conducted using a per-protocol population, including only participants who completed the assigned intervention and had available outcome data at the corresponding time points. Missing data were not imputed, and analyses were performed using complete-case analysis.

For repeated measurements across time points (W0, W4, W8, and W12), 2-way repeated-measures ANOVA was applied with treatment group as the between-subject factor and time as the within-subject factor, followed by appropriate post hoc comparisons when applicable. This approach was chosen due to the relatively small sample size, balanced group design, and limited number of predefined assessment time points. Two-tailed tests of statistical significance were used and performed using SPSS 23.0 (IBM, Armonk, NY, USA), with the significance level set at *P* <.05.

## 3. Results

### 3.1. Baseline demographics and characteristics of participants

**Figure 1.**
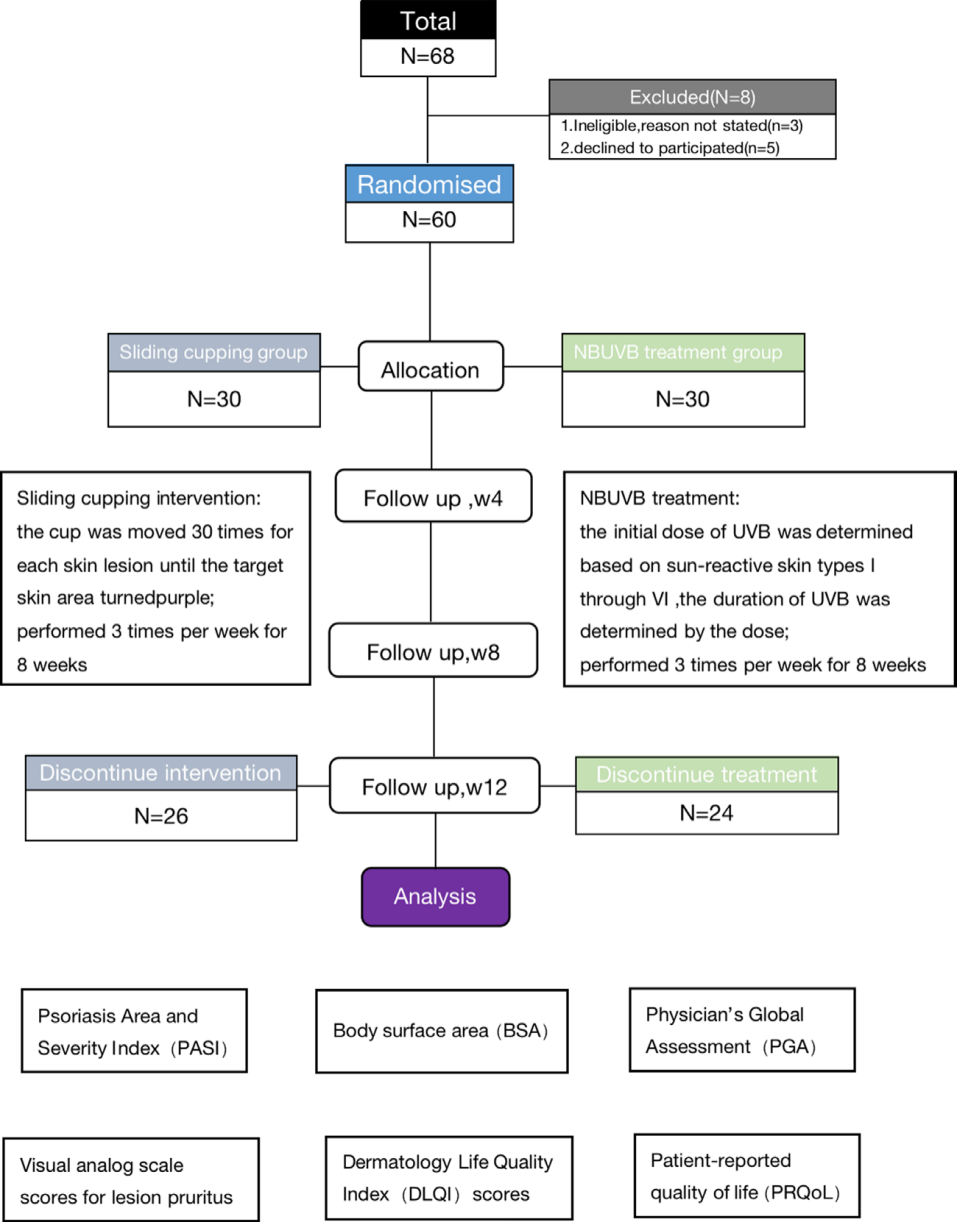
The diagram of consolidated standards of reporting trials.

**Figure 2.**
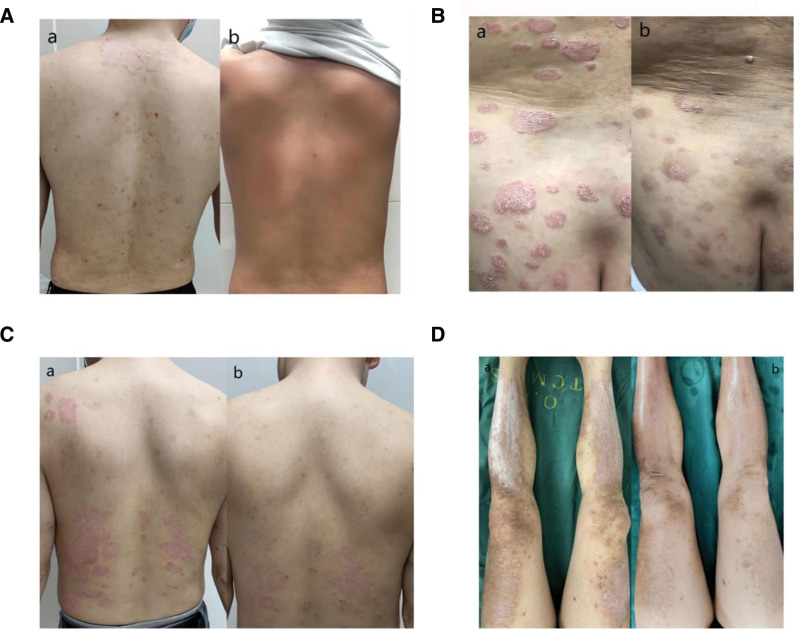
The representative skin appearance of NBUVB (A and B) and sliding cupping group (C and D). All the figures marked by lowercase “a” or “b” indicate the skin appearance before or after treatment, respectively. NBUVB = narrowband ultraviolet B.

A total of 68 patients with plaque psoriasis were consecutively included into this study and 8 patients were excluded according to the exclusion criteria (Fig. 1). Finally, 60 patients with plaque psoriasis receiving either sliding cupping intervention (n = 30) or NBUVB treatment (n = 30) for 8 weeks were included in this study. The representative skin appearance of participants is presented in Figure 2. The demographics and disease characteristics of participants at baseline were summarized in Table 1. Patient’s age, gender distribution, age of onset, psoriasis duration, BMI, the proportion of hypertension, current smoking, current drinking, previous psoriasis medication (vitamin D3 derivatives, traditional/herbal medicines, glucocorticoids, and moisturizers), PASI, BSA, PGA, VAS scores for lesion pruritus, DLQI, and PRQoL did not show significant difference across treatment

**Table 1:** Participant baseline demographics and characteristics.

Characteristic	Sliding cupping (n = 30)	NBUVB (n = 30)	*P*
Age (yr, mean ± s.d.)	40.17 ± 11.27	37.77 ± 12.68	.445
Gender (male, n/%)	23 (76.67%)	24 (80.00%)	.999
Psoriasis duration (yr, mean ± s.d.)	12.47 ± 8.19	11.53 ± 7.73	.652
Age of onset (yr, mean ± s.d.)	27.43 ± 8.97	25.93 ± 13.00	.605
BMI ≥24 (n/%)	19 (63.33%)	14 (46.67%)	.299
Hypertension (n/%)	2 (6.67%)	3 (10.00%)	.999
Family history (n/%)	14 (46.67%)	9 (30.00%)	.288
Current smoking (n/%)	17 (56.67%)	13 (43.33%)	.439
Current drinking (n/%)	11 (36.67%)	7 (23.33%)	.399
Previous psoriasis medication (n/%)	17 (56.67%)	24 (80.00%)	.095
Vitamin D3 derivative	7 (23.33%)	9 (30.00%)	–
Traditional/herbal medicine	8 (26.67%)	17 (56.67%)	–
Glucocorticoid	2 (6.67%)	7 (23.33%)	–
Moisturizers	5 (16.67%)	9 (30.00%)	–
PASI (mean ± s.d.)	6.34 ± 3.34	7.33 ± 3.35	.253
BSA (median, 25%–75%)	10.75 (3.50, 13.74)	12.99 (7.75, 14.50)	.333
PGA (median, 25%–75%)	2.00 (2.00, 3.00)	2.00 (2.00, 2.75)	.216
VAS scores for lesion pruritus (mean ± s.d.)	50.17 ± 24.93	41.33 ± 24.14	.169
DLQI (median, 25%–75%)	8.50 (5.00, 13.75)	7.50 (5.00, 9.00)	.347
PRQoL (median, 25%–75%)	10.50 (5.00, 13.50)	8.00 (3.75, 12.00)	.554

BMI = body mass index, BSA = body surface area, DLQI = dermatology life quality index, NBUVB = narrowband ultraviolet B, PASI = psoriasis area and severity index, PGA = physician’s global assessment, PRQoL = patient-reported quality of life, VAS = visual analogue scale.

### 3.2. Primary efficacy endpoint

The remaining participants were lost to follow-up before the 8-week assessment due to withdrawal of consent, poor treatment adherence, or inability to attend scheduled visits. No withdrawals were related to treatment-emergent AEs. Therefore, total response rates were calculated based on patients who completed the 8-week efficacy assessment (per-protocol population). Among patients receiving NBUVB treatment, 24 cases (80.00%) were successfully followed up. After 8 weeks of sliding cupping intervention, excellent response was achieved in 7 patients, good response was achieved in 11 patients, and no response was found in 8 patients. After 8 weeks of NBUVB treatment, excellent response was achieved in 8 patients, good response was achieved in 11 patients, and no response was found in 5 patients. The total response rates were 69.23% and 79.17% for patients receiving sliding cupping intervention and those receiving NBUVB treatment, respectively, and no statistically significant difference was observed between groups in this study (*P* = .526, Table 2). The representative appearance of both sliding cupping group and NBUVB group is shown in Figure 1.

**Table 2:** The total response rates for patients receiving sliding cupping intervention and those receiving NBUVB treatment.

Groups	Total response rate	*P*-value
Sliding cupping group	69.23%	.526
NBUVB group	79.17%	–

NBUVB = narrowband ultraviolet B.

### 3.3. Secondary efficacy endpoints

**Figure 3.**
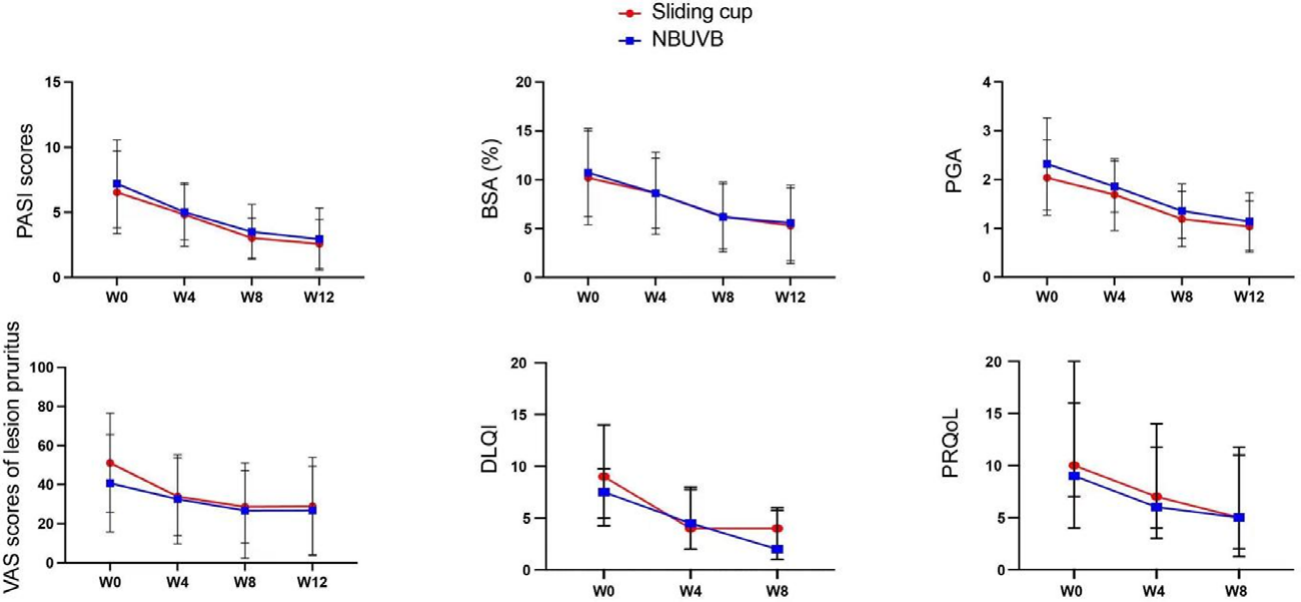
The PASI scores, BSA, PGA, VAS scores of lesion pruritus, DLQI, and PRQoL were reduced in patients with plaque psoriasis at different time points after either sliding cupping intervention or NBUVB treatment. BSA = body surface area, DLQI = dermatology life quality index, NBUVB = narrowband ultraviolet B, PASI = psoriasis area and severity index, PGA = physician’s global assessment, PRQoL = patient-reported quality of life, VAS = visual analogue scale.

It was found that the PASI scores, BSA, PGA, degrees of erythema, infiltration, and desquamation, and VAS scores of lesion pruritus were reduced in patients with plaque psoriasis at W0, W4 and W8 after either sliding cupping intervention or NBUVB treatment (*P* <.001). When the patients were followed up for further 4 weeks after 8-week treatment, their PASI scores, BSA, PGA, degrees of erythema, infiltration, and desquamation, and VAS scores of lesion pruritus were also declined (*P* <.001, Fig. 3). As presented in Figure 3, these secondary outcomes demonstrated consistent within-group improvements over time with comparable magnitudes of change between treatment groups, without statistically significant between-group differences at any assessment point. Patients receiving sliding cupping intervention and those receiving NBUVB treatment did not show statistically significant differences in these outcomes at W0, W4, W8, and W12 (*P* >.05). The DLQI scores were reduced in patients with plaque psoriasis at W0, W4 and W8 after either sliding cupping intervention or NBUVB treatment (*P* <.001), with no statistically significant differences observed between the 2 groups at these time points. The PRQoL scores did not significantly change in patients at W0, W4 and W8 after either sliding cupping intervention or NBUVB treatment, and no significant between-group differences were detected at W0, W4 and W8 (Fig. 3).

### 3.4. Safety evaluation

No significant differences in the frequency or severity of AEs were identified between the sliding cupping intervention and NBUVB treatment groups during the study period. A patient developed skin redness and pruritus after the first time of applying liquid paraffin during the sliding cup intervention, and these symptoms disappeared after 1 day. No adverse reactions occurred after the second time of applying liquid paraffin, which may be explained by temporary intolerance of the first time of sliding cup intervention. The skin redness, desquamation and slight tingling occurred in 2 patients after the dose increased to 2000 mJ/cm^2^, and these symptoms disappeared 3 to 4 days after withdrawal of NBUVB followed by use of moisturizers. No adverse reactions occurred after second treatment with 2000 mJ/cm^2^. There were 3 patients showing desquamation among patients receiving sliding cupping intervention, including 1 during the treatment and 2 during the follow-up. There were 2 patients showing desquamation during the treatment among patients receiving NBUVB treatment. The results of vital signs, routine blood tests, blood biochemistry, and routine urine tests of the overall population were normal. A summary of AEs by type, severity, and relationship to treatment is presented in Table 3.

**Table 3:** Summary of adverse events during the study period.

Adverse event	Severity	Relationship to treatment	Sliding cupping (n = 30)	NBUVB (n = 30)
Any adverse event	–	–	4 (13.33%)	2 (6.67%)
Skin redness	Mild	Related	1 (3.33%)	2 (6.67%)
Pruritus	Mild	Related	1 (3.33%)	0
Desquamation	Mild	Related	3 (10.00%)	2 (6.67%)
Tingling/burning sensation	Mild	Related	0	2 (6.67%)
Serious adverse events	–	–	0	0
Treatment discontinuation due to AE	–	–	0	0

AE = adverse events, NBUVB = narrowband ultraviolet B.

## 4. Discussion

Although there have been multiple therapies for the treatment of psoriasis, while multi-treatment failure usually occurs in the clinical practice.^[31]^ Plaque psoriasis is a refractory disease, and it is urgent to develop more effective methods or combination therapies. Cupping therapy has been widely used in treating many health disorders since ancient time, but its utilization in the treatment of psoriasis has been seen in only small-scale clinical studies. In this investigation, we designed a group of plaque psoriasis patients receiving NBUVB treatment as control and analyzed efficacy and safety of sliding cupping therapy in treating plaque psoriasis. The overall results did not show significant differences in efficacy or safety outcomes between sliding cupping therapy and NBUVB treatment in this small, short-term trial, without implying formal equivalence or non-inferiority. The following mechanistic considerations are primarily based on previous experimental and clinical research and should be interpreted as hypothesis-generating rather than mechanisms directly demonstrated by the present clinical trial.

Psoriasis is an immune-mediated disorder, in which the immune response is characterized by T cells, neutrophils and macrophages infiltration into dermal and epidermal layers of the skin.^[32]^ Th17 T cells are implicated in the pathogenesis of psoriasis by triggering dendritic cells and keratinocytes, further resulting in an increased release of inflammatory cytokines and upregulation of chemokines. The therapeutic effects of sliding cupping treatment may be mediated for its effect on regulation of the T subsets of lymphocytes and inhibition of inflammatory response.^[33]^ Although alterations in cytokine signaling, immune cell subsets, and metabololipidomic profiles have been reported in previous studies of cupping therapy, these biological effects were not directly measured in the current study and therefore cannot be causally linked to the observed clinical outcomes. Psoriasis is also significantly associated with lipid metabolism.^[34]^ After receiving sliding cupping treatment, the patients with psoriasis exhibited increased expressions of anti-inflammatory lipids, such as PGE1, 5,6-EET and 14,15-EET, while the production of pro-inflammatory metabololipidome, such as 12-HETE and TXB2, was reduced in the skin and plasma.^[35]^ Accordingly, PGE1 and 5,6-EET reduced the level of TNF-α, whereas 5,6-EET and 5,6-DHET reduced the production of IL-6 in macrophages. In addition, cupping can reduce the blood content of superoxide dismutase, thereby inhibiting oxidative stress.^[36,37]^ The cupping therapy not only significantly elevates the level of oxyhemoglobin but also declines the content of hemoglobin at the treatment site after cupping.^[38]^ Besides, cupping therapy can significantly relieve the degree of pruritus in chronic spontaneous urticaria via decreasing the level of IgE and IL-4, and these had been shown to be involved in mechanisms of itch in Psoriasis.^[39]^ The sliding cupping therapy can modulate immune function and thus enhance skin tolerance due to its combination of warm moxibustion, massage, scraping, as well as drug therapy.^[40,41]^ In addition, with the aid of lubricants, the skin barrier function of patients was significantly improved, thus a certain drug treatment effect was achieved to reinforce the sliding cupping therapy.^[42]^ Although a clear mechanism behind sliding cupping for treating psoriasis remains less understood, our data combined with other investigations yielded an indication that sliding cupping can attenuate the local inflammation over the skin lesion and alleviate excessive thickening of the plaque psoriatic skin. Thus, any proposed immunomodulatory or metabolic effects of sliding cupping in psoriasis should be regarded as putative mechanisms requiring confirmation in future mechanistic and biomarker-driven studies.

Several important limitations of this study should be acknowledged. Firstly, this was a single-center trial with a relatively small sample size, which might limit statistical power and increase the risk of type II error. Secondly, the study population consisted predominantly of Chinese patients, which might limit the generalizability of the findings to other ethnic or geographic populations. Thirdly, despite standardized assessment procedures, the study was not fully blinded, and this might introduce assessment or performance bias. Fourthly, the follow-up time was limited to 12 weeks, and long-term efficacy, relapse rates, and durability of response could not be evaluated. Fifthly, sliding cupping therapy is inherently operator-dependent, and variations in technique, applied pressure, and cup size might influence treatment consistency and outcomes. Moreover, no formal cost-effectiveness analysis was performed, precluding conclusions regarding the economic feasibility of sliding cupping compared with standard phototherapy. Finally, baseline PASI scores in this cohort were relatively low for a population often described as having moderate-to-severe psoriasis; therefore, the results might not be generalizable to patients with more severe disease or extensive BSA involvement.

In conclusion, the present study offers more evidence-based data on the sliding cupping therapy, this ancient Chinese medical therapy, exhibits statistically similar efficacy and safety profiles as NBUVB treatment, which may facilitate decision making regarding plaque psoriasis treatment. From a practical perspective, implementation of sliding cupping therapy in routine clinical practice requires trained practitioners who can perform the procedure with consistent technique and appropriate force. In contrast to the standardized, device-based nature of NBUVB phototherapy, sliding cupping is manually applied and therefore more susceptible to intra- and inter-operator variability. This variability in application might influence both treatment reproducibility and patient experience, and could present challenges for widespread adoption in settings with limited availability of trained personnel. Conversely, where expertise in traditional manipulative therapies is readily accessible, sliding cupping may represent a feasible complementary option that integrates into existing dermatologic care. Sliding cupping therapy may serve as a potential alternative strategy for plaque psoriasis. Future investigations should concentrate on long-term effects of the sliding cupping therapy on psoriasis recurrence and whether sliding cupping therapy as an alternative strategy could prevent the incidence of pigmentation caused by phototherapy, as well as potential skin tumors.

## Author contributions

**Conceptualization:** Jia Liu, Shun Guo.

**Data curation:** Jia Liu, Shun Guo.

**Formal analysis:** Yue Shi.

**Funding acquisition:** Shun Guo.

**Investigation:** Cheng Tan.

**Methodology:** Jing Tan, Yue Shi, Cheng Tan.

**Project administration:** Cheng Tan.

**Resources:** Miao Miao.

**Software:** Jing Tan.

**Supervision:** Miao Miao, Cheng Tan, Yuegang Wei.

**Validation:** Jing Tan, Miao Miao.

**Visualization:** Yue Shi.

**Writing – original draft:** Jia Liu, Shun Guo.

**Writing – review & editing:** Yuegang Wei.
